# The Use of a Single Trunk-Mounted Accelerometer to Detect Changes in Center of Mass Motion Linked to Lower-Leg Overuse Injuries: A Prospective Study

**DOI:** 10.3390/s21217385

**Published:** 2021-11-06

**Authors:** Gerard Aristizábal Pla, Enzo Hollville, Kurt Schütte, Benedicte Vanwanseele

**Affiliations:** 1 Human Movements Biomechanics Research Group, Department of Movement Sciences, KU Leuven, 3001 Leuven, Belgium; garistizabal@umass.edu (G.A.P.); Enzo.HOLLVILLE@insep.fr (E.H.); Kurtschutte.sa@gmail.com (K.S.); 2 Department of Kinesiology, UMASS Amherst Amherst, University of Massachusetts Integrative Locomotion Lab, Amherst, MA 01003, USA

**Keywords:** accelerometer, running, fatigue, lower-leg overuse injury

## Abstract

Movement dynamics during running was previously characterized using a trunk-mounted accelerometer, and were associated with a history of overuse injuries. However, it remains unknown if these measures are also linked to the development of overuse injuries. The aim of this study was therefore to determine how movement dynamics alter in response to fatigue, and the possible link with developing lower-leg overuse injuries during a six-month follow-up period. Two hundred and eight movement science university students completed a 12-min all-out run while wearing a trunk-mounted accelerometer. Dynamic stability, dynamic loading and spatiotemporal measures were extracted from the accelerometer. Participants sustaining an injury within the 6-month period demonstrated significantly higher RMS ratio values in the vertical direction and lower RMS ratio values in the anteroposterior direction, and lower impact acceleration values in the anteroposterior direction in an unfatigued state compared to the uninjured group. They also demonstrated an increase in dynamic loading in the horizontal plane during the run. In addition, with running fatigue both groups exhibited changes in dynamic stability and loading measures. These results show the potential of using a single trunk-mounted accelerometer to detect changes in movement dynamics that are linked to lower-leg overuse injuries.

## 1. Introduction

Running-related injuries (RRIs) are common, with incidence rates going from 6.8 to 59 injuries per 1000 h of running [1,2,3,4,5,6]. This represents a socio-economic burden, resulting in healthcare utilization and loss of productivity costs [6], and could lead to large consequences such as physical inactivity with its respective associated risks. RRIs are mostly considered as overuse injuries resulting from repetitive loading of the musculoskeletal system. Previous studies investigating their multifactorial etiology reported several biomechanical factors linked to overload injury, such as the magnitude and duration of load applied to the human body, as well as the load distribution among different internal structures [7,8]. However, most of these studies are retrospective, meaning that they have a limited capacity to conclude if those factors precede the development of RRIs, or if they are the consequences of injury [7].

Current knowledge about the development of RRIs is mostly founded on laboratory-based investigations using three-dimensional motion capture combined with ground reaction forces to establish running patterns that put runners at risk. However, this type of analysis only provides a controlled snapshot of the running style, e.g. one moment in time, in an artificial controlled environment, which might not represent the natural running pattern or changes in running pattern during a training session.

The recent availability of wearable accelerometers that can measure changes in movement dynamics during running in the ecological environment of the runner allowed the first steps towards providing a completer picture of how runners behave during a training session, adapting their pattern to fatigue, or potentially prior to the onset of injury. Previous research showed that wearable trunk accelerometers can successfully identify movement deviations caused by running fatigue in indoor [9] and outdoor running conditions [10,11,12,13,14]. In addition, a very recent study by Winter et al. demonstrated the potential of wearable trunk accelerometers to identify movement deviations prior to injury occurrence [11,12]. Lightweight wearable devices present a lot of advantages, such as out of the lab gait analysis and the possibility of providing real-time feedback—in combination with the correct movement dynamics parameter extraction, this can potentially be used to make a training adjustable based on individual movement dynamics.

Several biomechanical meaningful parameters can be extracted from the acceleration signal, including root mean square (RMS) ratio, regularities between steps and strides, sample entropy and peak accelerations. Regularity is defined as the similarity of the tri-axial trunk acceleration signal comparing consecutive steps or strides. These measures can be classified as dynamic stability or loading measures [9,10]. Dynamic stability represents the runners’ capability of maintaining an optimal variability, uniformity, regularity and complexity of the acceleration signal. Dynamic stability decreases with fatigue accumulation during running, predominantly through an increase in variability (RMS ratio) in the horizontal plane (i.e., sway) [9,10,11,14]. In addition, a larger decrease in dynamic stability with running fatigue was observed in participants with a history of medial tibial stress syndrome compared to controls [10]. Dynamic loading represents the magnitude of impacts on the human body during ground contact while running [15]. An increase in dynamic loading in the anterior-posterior direction during a fatiguing protocol has been observed prior to the onset of RRIs [12]. Unfortunately, only one prospective study [11,12] has investigated the link between accelerometry-based measures of dynamic stability and dynamic loading to the development of RRIs. In this one-year prospective study [11,12], differences between injured and uninjured runners in running dynamics were only investigated at the end of a fatiguing run. Winter et al. [12] reported that both injured and uninjured runners increased the loading in the horizontal plane, but injured runners increased their loading to a greater extent. It remains unknown when these changes in movement dynamics start, and if they can be identified earlier in the fatiguing protocol. Early detection could potentially avoid unnecessary stresses and strains to musculoskeletal tissues with the accumulation of fatigue. Therefore, more prospective studies examining differences in running dynamics during a fatiguing protocol are needed to better appraise the relationship between RRIs and accelerometry measures.

The main aim of this study is to investigate accelerometry based-measures of dynamic stability, and loading as potential risk factors for the development of lower-leg overuse injuries (LLOI). We therefore investigate how these parameters may change in response to fatigue, and their possible link with developing lower-leg overuse injuries. We hypothesize that fatigue will mainly decrease dynamic stability in the horizontal plane. In addition, we hypothesize that participants who sustain an injury during a follow-up period of 6 months will exhibit a larger decrease in dynamic stability and a higher increase in dynamic loading during a fatiguing protocol, compared to subjects who remained uninjured. We specifically hypothesize that: (i) based on findings of our previous study [9,10], fatigue will cause an increase of the RMS ratio in the horizontal plane and a decrease in regularities between steps in the vertical direction; and (ii) based on the findings of [10,12], participants who sustain an injury will have a larger increase in RMS ratio in the horizontal plane and a higher increase of the impact acceleration in all directions during the fatigue running protocol, compared to the uninjured group.

## 2. Materials and Methods

### 2.1. Participants

Two-hundred and eight first year undergraduate movement science university students (144 male, 64 female) from two separate first year cohorts (2018–2019, 2019–2020) at KU Leuven (Belgium) were enrolled to participate in this study. All participants provided written informed consent before participation in accordance with the Declaration of Helsinki, and were screened to know their health condition on the test day. The local ethics committee of University Hospital Leuven approved this study (S60810).

All students visited a sport medicine physician at the Sport Medical Advise Center (University Hospital Leuven) for a medical screening including recording of the injury history. All students followed the same academic sports program at a common sport facility for 26 weeks per academic year. Sports included soccer, handball, basketball, volleyball, track and field, gymnastics, dance and swimming. The weekly program consisted of 10 h of sports on average. Students were required to report all injuries to the sport medicine physician of the Sport Medical Advise Center (University Hospital Leuven). In consultation with the sports medicine physician, a LLOI was defined as an overuse injury of the lower extremities occurring during physical activity and having at least one of the following consequences: (1) a reduction in the amount or level of sports activity; or (2) a need for (medical) advice or treatment. All injury data were captured in a standardized injury report that recorded the type of injury and treatment of the injury.

### 2.2. Experimental Protocol

**Figure 1:**
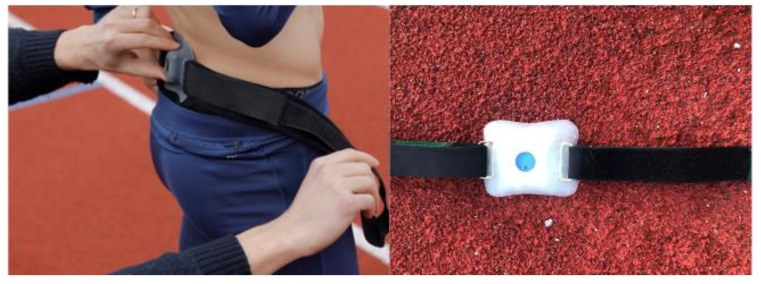
Sensor in the custom-designed neoprene pocket, tightly secured within a waist belt.

During the first week of the academic year, participants performed a 12-min all-out run on a typical 400 m synthetic track in their own running shoes (University Sport Center, KU Leuven). Participants were asked to run as far as possible at a steady pace for 12 min. Measurements took place in four sessions on two different days. The sessions were completed under slightly different weather conditions, but with an average temperature of 17 °C. At the start of the session, an accelerometer (±16 g range, sampling at 1000 Hz, 16-bit resolution, mass = 5 g; Byteflies, Antwerp, Belgium) was securely positioned at the lower trunk level, over the L3-L5 spinous process of the lower back using a custom-designed neoprene pocket, tightly secured within a waist belt (Figure 1).This sensor location was chosen based on results from our previous study [10], where we observed changes with fatigue and injury at the trunk level and not at the ankle. Before the 12-min run, all participants performed a standardized warm-up, including running two laps (800 m), and performing some drills such as skipping, sprints and stretching. At the end of the run, the running distance covered was recorded for each participant.

### 2.3. Accelerometry Processing

The raw acceleration signals were downloaded from the sensors and converted to a GUI readable format using customized software in MATLAB version R2018b (The Mathworks Inc., Natick, MA, USA). The accelerometers continuously recorded accelerations during all sessions on that day, so we manually divided the signals into individual sessions allocated to individual participants. A quality check was performed on the signal checking: (1) if the signal recorded exceeded the range of the sensor (16 g); (2) if the sensor was upside down during the recordings; or (3) if signals did not have 12 min of running data (participants stopped during the run). Signals that exceeded the range or did not have 12 min of running data were excluded, while inverted signals were corrected.

The processing of all tri-axial trunk accelerations was performed using customized software in Python (Python Software Foundation, Delaware, DE, USA). To ensure we only included steady state running, the first 10% of the 12 min run was removed. Because of lumbar curvature and global trunk lean while running, it was expected that the sensor coordinate system was not fully aligned with the global coordinate system. Therefore, a commonly used tilt correction procedure proposed by Moe-Nilssen et al. [16] was applied to all directions before the extraction of accelerometry-based features. Then, a step detection method based on the automated algorithms proposed by Benson et al. [17] was used to identify initial contacts and toe-offs. The algorithm was modified slightly to account for the specification of our sensor. After carefully checking the results of the algorithm, we decided on the following procedure: the vertical and anteroposterior signals were preprocessed with a zero-lag low-pass filter (Butterworth, 4th order, 8 Hz of cut-off frequency). Then, a sliding window approach was used to obtain gait events. Gait events were determined as minimum peaks or values in the AP signal between two consecutive VT peaks. Next, dynamic stability, dynamic loading and spatiotemporal measures were extracted from the three-dimensional accelerations (vertical, VT, mediolateral, ML and anteroposterior, AP).

Dynamic stability measures were computed for all the three directions. Firstly, the acceleration RMS ratio was calculated [10]. The VT, ML and AP acceleration RMS constitutes the absolute magnitude of the variability in each direction. The RMS ratio is calculated by normalizing each RMS with the resultant vector RMS. Thus, the acceleration RMS ratio indicates how each acceleration direction contributes proportionally to the whole movement variability. Secondly, we calculated the regularity of steps and strides using the primary and secondary dominant unbiased autocorrelation coefficients to indicate consistency between steps and strides [18]. A value of 1 indicates a perfect step/stride regularity. Note that for the extraction of the RMS ratio and the step and stride regularity a low-pass filter (Butterworth, 4th order, 50 Hz of cut-off frequency) was applied to the tri-axial trunk accelerations. Finally, we calculated sample entropy as a non-linear measure to capture complexity of unfiltered acceleration waveforms, with values typically within ranges of 0–2 for physiological systems. The higher the values of the sample entropy, the less periodic or more unpredictable the running gait is.

**Figure 2:**
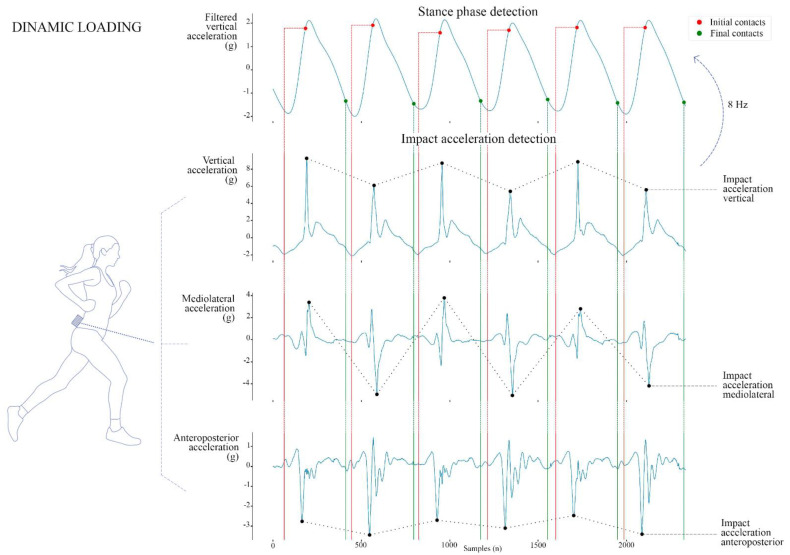
Extraction of the impact acceleration. Dotted lines represent the windows during which impact peaks were detected. Vertical impact acceleration was positive, as it reflected the contributions of the impact peak in the vertical ground reaction forces. Mediolateral impact acceleration was extracted using the absolute value, as it was positive and negative for right and left steps respectively. Anteroposterior impact acceleration was negative as it corresponded to the braking phase of running. Initial and final contacts were obtained using the step detection method proposed by Benson et al. [12], which preprocessed the vertical signal with a low-pass filter.

Dynamic loading was extracted using the peak acceleration during stance phases in all three directions from the unfiltered tri-axial trunk accelerations (Figure 2). Peak VT acceleration was positive as it reflected the contributions of the impact peak in the vertical ground reaction forces. Peak acceleration ML was extracted using the absolute value, as it was positive and negative for right and left steps respectively. Peak acceleration AP was negative, as it corresponded to the braking phase of running.

Spatio-temporal measures were extracted from the acceleration signals. Specifically, contact time was calculated as the time between initial contact and toe-off for each step using the step-detection algorithm [17]. Step frequency was calculated based on the unbiased autocorrelation signal in the 50 Hz filtered VT acceleration signal, using samples per dominant period of the autocorrelation peak and sampling frequency of the accelerometer as inputs [10,18].

All values for the dynamic stability, dynamic loading and spatio-temporal measures were averaged for each 10% interval of the 12-min all-out run, resulting in a single value for each measure obtained during that time interval. Since we removed the first 10% of the run due to non-steady pace, nine intervals (i.e., nine data points) were obtained during the run for each variable.

### 2.4. Statistical Analyses

Before the statistical analysis, all subjects were assigned to two groups: a group who developed an injury (injured group); and a group who did not (uninjured group) during the six months follow-up. SPSS (version 26; SPSS Inc., Chicago, IL, USA) was used for all statistical examinations. After normal distribution was confirmed for all measures (−1 < accepted skewness < 1), changes between repeated measures (10% intervals) during the run were assessed using the generalized estimating equations method. In particular, an exchangeable correlation structure was specified, since repeated measures per subject were correlated. The generalized estimating equations method used a mixed-between-within participants design, combining the within-participants design and the between-participants design. The within-participants design was used to examine changes in the continuous dependent variables (RMS ratio, step regularity, stride regularity, sample entropy, impact acceleration, contact time and step frequency) measured over time. The mixed-between-within participants design also examined whether these changes were significantly associated with a categorical independent grouping variable (sustaining an overuse injury) — this is called the interaction effect. To test group differences at baseline (first interval of the steady-state run; 10–20%), a one-way ANOVA (for normally distributed measures) or a nonparametric Mann-Whitney U test (for not normally distributed measures) were used. Effect sizes were calculated and reported as partial eta squared (η^2^_p_) with η^2^_p_ = 0.01 being a small effect, η^2^_p_ = 0.06 being a medium effect and η^2^_p_ = 0.14 being a large effect. To analyze differences between groups for descriptive characteristics, the independent t-test was used. Alpha level was set to 0.05 for all analyses.

## 3. Results

One hundred and thirty-two participants were included in the analysis. Data of 9 participants were missing due to the malfunctioning of the sensor during the run. 14 additional participants were excluded because of incomplete runs and 47 were excluded due to bad signal quality (exceeded range, drop out of the signal or sudden peaks or drops in the signal). In addition, 6 participants dropped out of the academic program, so no injury data was available.

During the six months follow-up, 33% of the participants sustained a LLOI. Descriptive characteristics for all included participants, as well as for the injured and uninjured group, are listed in Table 1. No significant differences were found for body mass, height, BMI or performance between the injured and the uninjured group.

LLOIs were grouped as muscle injury, patellofemoral pain, iliotibial band injury, medial tibial stress syndrome (MTSS), ankle pain and bone overuse injury. The distribution of the type of injuries sustained by the injured group is presented in Table 2. The most common injury was the MTSS, compromising 40% of all injuries.

### 3.1. Differences between Injured and Non-Injured Participants at Baseline

We found a main group effect at baseline for the RMS ratio in the VT and AP directions and for the impact acceleration in the AP direction (Table 3). The injured group exhibited a higher RMS ratio in the VT direction, a lower RMS ratio in the AP direction and lower impact acceleration values in the AP direction compared to the uninjured group.

### 3.2. Fatigue Effect on Dynamic Stability, Loading and Spatiotemporal Measures

**Figure 3:**
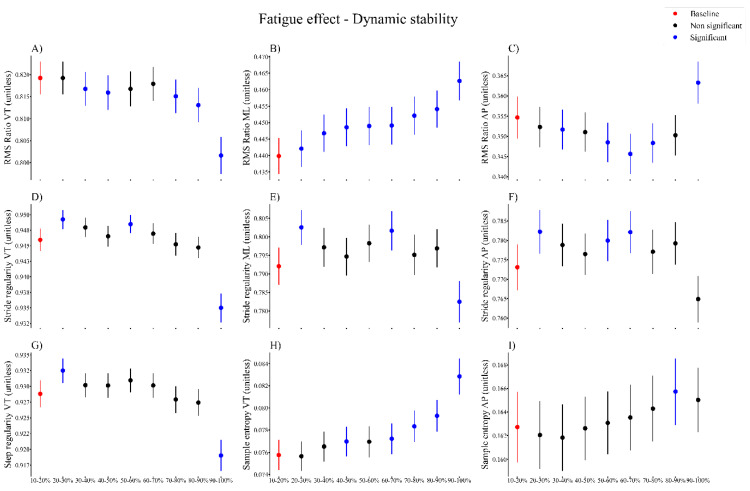
Time series of the overall significant fatigue effects (represented by blue dots) for dynamic stability measures ((**A**–**C**) RMS Ratio in vertical, mediolateral and anteroposterior direction; (**D**–**F**) Stride regularity in all 3 directions; (**G**) step regularity in the vertical direction; (**H**–**I**) Sample entropy in the vertical and anteroposterior direction) for all participants, compared to baseline (represented by red dots). VT, vertical, ML, mediolateral, AP, anteroposterior.

Dynamic stability (RMS ratio, step regularity, stride regularity and sample entropy) changed overall during the fatiguing run (Figure 3). Compared to the baseline value, the RMS ratio in the VT direction decreased from 30% until 50%, and from 70% until the end of the run (Figure 3A). The RMS ratio in the ML direction increased from 20% until the end of the run (Figure 3B). The RMS ratio in the AP direction decreased from 30% until 40%, from 50% until 80%, and it increased from 90% until the end of the run (Figure 3C). There was an effect of fatigue on stride regularity in all three directions (Figure 3D–F). Stride regularity in the VT direction increased from 20% until 30%, and 50% until 60%. From 90% until the end of the run (Figure 3D), stride regularity was decreased compared to the baseline. We also found an increase in the stride regularity in the ML direction from 20% until 30%, from 60% until 70%, and from 90% until 100%, and in the AP direction from 20% until 30%, and from 50% until 70% (Figure 3E,F) compared to baseline. There was an overall effect of fatigue on step regularity in the VT direction (Figure 3G). Step regularity in the VT direction increased early during the running protocol (at 20–30%) and decreased at the end of the run (at 90% until the end) compared to the baseline. There was an effect of fatigue on sample entropy in the VT and AP directions. Sample entropy increased from 40% until 50% and 60% until the end of the run in the VT direction (Figure 3H), and increased at 80% until 90% of the run in the AP direction (Figure 3I) compared to the baseline.

**Figure 4:**
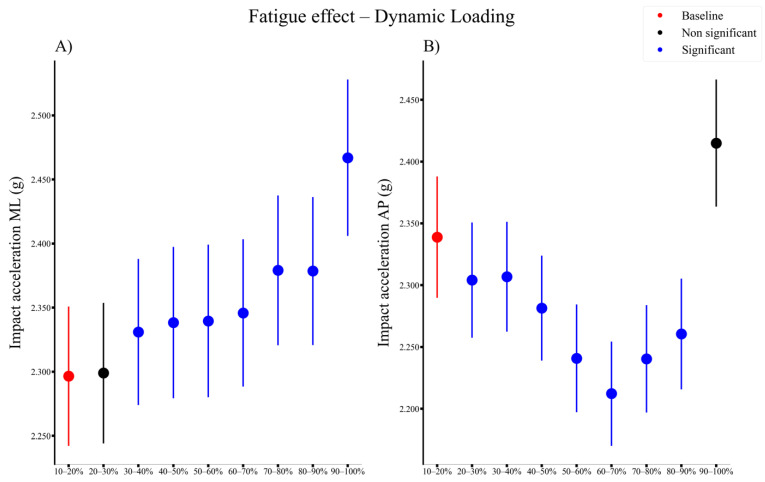
Time series of the overall significant fatigue effects (represented by blue dots) for dynamic loading measures ((**A**) Peak impact acceleration in the mediolateral direction and (**B**) peak impact acceleration in the anteroposterior direction) for all participants, compared to the baseline (represented by red dots). ML, mediolateral, AP, anteroposterior.

There was a significant main fatigue effect on the dynamic loading (peak acceleration in ML and AP direction). In the ML direction, impact acceleration increased significantly from 30% until the end of the run (Figure 4A), while in the AP direction, impact acceleration decreased significantly from 20% until 80% of the run (Figure 4B) compared to the baseline.

**Figure 5:**
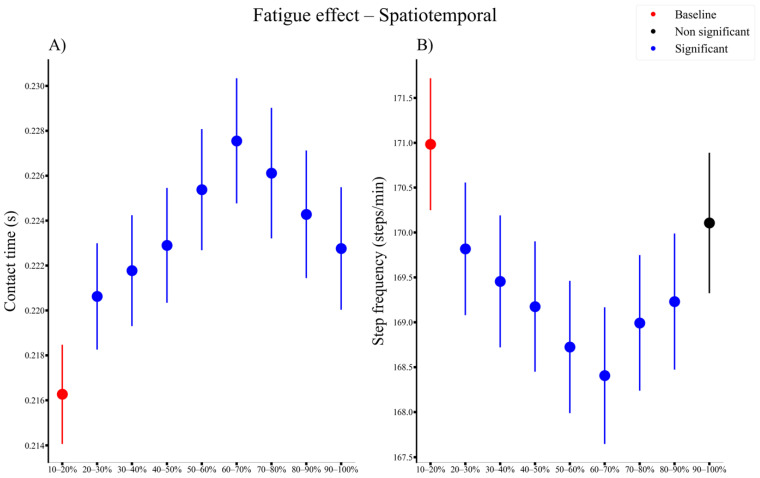
Time series of the overall significant fatigue effects (represented by blue dots) for spatiotemporal measures ((**A**) contact time and (**B**) step frequency) for all participants compared to the baseline (represented by red dots). VT, vertical.

There was a significant main fatigue effect for all spatiotemporal parameters (Figure 5). The contact time increased from 20% until the end of the run (Figure 4A), while in the meantime, step frequency significantly decreased from 20% until 90% (Figure 5B) compared to the baseline.

### 3.3. Interaction Effect on Dynamic Stability, Loading and Spatiotemporal Measures

**Figure 6:**
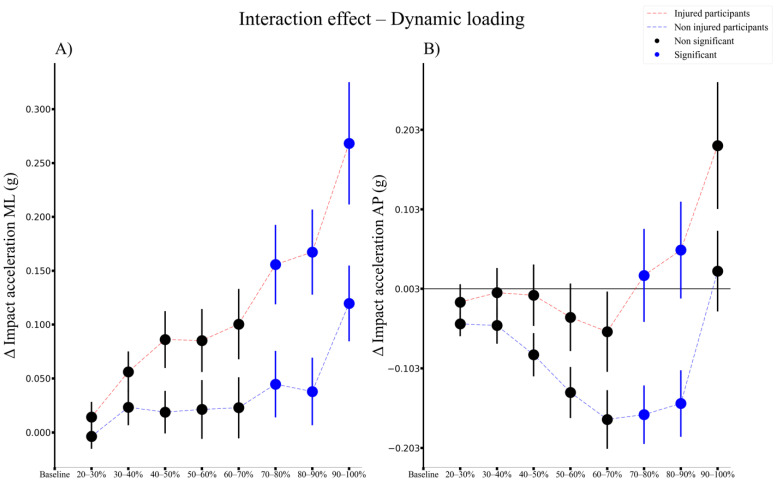
Time series of the overall significant interaction effects (represented by blue dots) for changes in dynamic loading measures (Change from baseline in peak impact acceleration in (**A**) mediolateral direction and (**B**) anterioposterior direction) for subjects that sustained an injury (red dashed lines) and subjects that would not (blue dashed lines), with differences relative to the baseline (first percent of the Cooper test). ML, mediolateral, AP, anteroposterior.

The peak impact accelerations in the ML and AP directions exhibited a significant fatigue by group interaction effect (Figure 6). With the accumulation of running fatigue, the injured group exhibited a larger increase in the impact acceleration in the ML direction between 70 and 90% of the run (Figure 6A), and an increase in the AP direction during 70 and 80% of the run (Figure 6B) compared to the uninjured group. When including the BMI and the performance parameter as covariates, the only interaction effect that changed was the impact acceleration in the ML direction, which was only significant in 80% of the run compared to the baseline.

## 4. Discussion

The aim of this study was to investigate how accelerometry-based features changed in response to fatigue and their association with the development of LLOI. This study found that during a fatiguing run, peak accelerations in the ML and AP directions increased more in participants who sustained an LLOI during the 6 months follow-up. We also confirmed that fatigue induces changes in dynamic stability and loading in outdoor running environments.

A trunk-mounted accelerometer was able to detect movement compensations during a running fatigue protocol. Furthermore, some of these compensations were associated with the development of LLOIs. These findings build further on limited available literature that reported movement compensations and changes in movement dynamics after a fatiguing running [9,10,11,12,14] and prior LLOI occurrence [11,12]. In addition, in contrast to our previous study [10], we did find differences between the injured group and the uninjured group during steady state unfatigued running. Thus, a trunk-mounted wearable accelerometer could be successful in identifying parameters in running dynamics linked with the development of LLOIs in both fatigued and unfatigued states.

In the unfatigued state, dynamic loading and dynamic stability were different between the injured and the uninjured group, although the effect sizes were small. The injured group ran with lower peak acceleration values in the AP direction compared to the uninjured group. The reduced AP peak acceleration could indicate lower braking force, and therefore a more efficient running pattern, although this could also be associated with a slower, more conservative way of starting the run. Unfortunately, we did not record speed during each lap to confirm this hypothesis, and although the average speed of the run was not significantly different between the injured and non-injured runners, there is still a trend toward a lower performance. Although previous accelerometer-based studies did not observe any difference between injured and non-injured runners [1,2,3], lab-based studies previously observed lower braking forces in runners with the presence of LLOIs (i.e., patellofemoral pain [19], iliotibial band syndrome [20]). Investigators also linked the lower peak braking force to a slower running pace. In contrast, one prospective study showed the opposite, i.e., higher braking forces in runners sustaining an injury after a follow-up period [21] compared to healthy runners. However, this latter study was performed on a treadmill, including only females running at a constant speed, which could explain the differences with our study results. Our injured group also exhibited a significantly higher RMS ratio in the VT and lower RMS ratio in the AP direction during an unfatigued steady state running compared to the non-injured group. These results seem to confirm previous literature linking a lower dynamic stability in the VT direction with LLOI occurrence for slow and intermediate runners [12]. In this one-year prospective study [11,12], differences between injured and uninjured runners in running dynamics were investigated during a long-distance run using a single trunk mounted accelerometer. In an unfatigued state, injured slow runners exhibited lower step regularities in the VT direction compared to the non-injured runners [12]. Together with the results of the current study, it seems that a lower dynamic stability in the VT could indicate a higher risk for developing LLOI. As the effect sizes of these measures are still small, further research is needed to confirm the role of dynamic stability in the development of LLOI.

In a fatigued state, dynamic stability in the ML direction decreased, step and stride regularity in the VT direction decreased and sample entropy in the VT increased for both groups. These findings are in line with previous investigations using a similar trunk-mounted accelerometer and accelerometer-based features [1,2,3,4]. In our previous study, using a similar fatiguing protocol with a similar cohort, we observed a decrease in the step regularity in the VT direction and an increase for the RMS ratio in the ML direction [3]. Winter et al. [11] did not find a decrease in step regularity, but did find a similar increase in RMS ratio in the ML direction in their male runners. In addition, Clermont et al. [14] found that a marathon race induced a prolongated fatigue state 2 days following the race, characterized by an elevated peak ML acceleration and an increased RMS ratio ML in comparison to the day prior to the marathon race. Together with the results from this study, evidence is growing that running fatigue results in an increased variability in the ML direction already early in the run (from 30% onwards), and that these changes even persist days after the fatiguing run. This increase in variability suggests a less efficient running style, as it has been linked to increases in postural sway and greater lateral trunk motion [5], and as accelerations in the ML direction do not contribute to forward running [6]. The higher variability in combination with the higher ML impact peaks at the end of the run could potentially expose musculoskeletal tissues to higher stresses and strains.

Running fatigue-induced changes in dynamic loading were different between the injured and the non-injured group. The injured group exhibited a larger increase in the peak acceleration ML and AP values than the uninjured group, with the accumulation of running fatigue. These findings build further on limited available evidence that observed an increase in dynamic loading prior to injury occurrence. Winter et al. [11] reported that both injured and uninjured runners increased peak acceleration in AP direction towards the end of an 8 km run. However, they observed that injured runners increased their peak AP acceleration to a greater extent. The current study observed a decrease for the uninjured group and an increase in the injured group with the accumulation of running fatigue with respect to baseline. This difference could arise from the difference in fatiguing protocol or the study cohort (e.g., runners vs. movement science university students). It seems that an increase in loading in the horizontal plane with running fatigue possibly occurs as a compensation strategy, which put runners at a higher risk for LLOIs, as the increased horizontal load does not contribute to forward running and could ultimately increase the loading on the musculoskeletal tissues. The higher peak ML and AP acceleration in the injured group could also be associated with hip and trunk kinematics, which have been previously associated with LLOIs (e.g., iliotibial band [22,23], patellofemoral pain [24]), as our accelerometers were placed close to the hip center. These changes in movement kinematics not only occur after [22,24], but also prior to onset of LLOI [23].

Although this study demonstrated the value of a single trunk-mounted accelerometer to monitor running fatigue and its potential to indicate an increased risk for LLOIs, these accelerometer-based features could have been sensitive to additional factors, such as foot strike pattern, running speed, running shoe selection and weather. In addition, LLOIs not only occur due to a specific running pattern, but training load and progression are also known to play a crucial role. Although our cohort followed a similar training program, some of the students still performed additional training in their free time, which could have contributed to the development of LLOIs. Furthermore, our study did not have enough power to investigate if they might be different gender-specific parameters. A larger sample could have possibly revealed sex or injury specific changes in movement dynamics, and could contribute further to the generalizability of our results.

In conclusion, the results of this study demonstrate the potential of using a single trunk-mounted accelerometer to detect changes in movement dynamics that are linked to lower-leg overuse injuries. Both dynamic loading and dynamic stability parameters could distinguish between the prospectively injured and the uninjured groups, as well as identify a fatigued state of running. In addition, runners who sustained an injury within 6 months were running with higher impact accelerations in the horizontal plane. Further research will need to focus on the potential to monitor these parameters during training sessions, and investigate if interventions can be developed to improve dynamic stability and loading.

## Figures and Tables

**Table 1:** Descriptive characteristics for all participants. Values are means ± SD.

Characteristics	All Participants (n = 132)	Lower Extremity Overuse Injury (n = 45)	No Injury (n = 88)
Sex (male/female)	96/42	27/18	64/23
Age (years)	18.7 ± 2.0	19.2 ± 2.9	18.4 ± 1.3
Body mass (kg)	67.8 ± 9.1	67.8 ± 10.6	67.9 ± 8.3
Height (m)	1.76 ± 0.08	1.75 ± 0.09	1.76 ± 0.08
Body mass index (kg/m^2^)	21.9 ±2.1	22.07 ± 2.6	21.8 ± 2.0
Running test performance (m)	2786 ± 382	2730 ± 413	2819 ± 362

**Table 2:** Type and incidence of lower limb overuse injury recorded during the follow-up 6 months.

Classification of Injury	Number of Participants
MTSS	18 (40%)
Muscle injury	16 (35.6%)
Ankle injury	4 (8.9%)
Patellofemoral pain	4 (8.9%)
Iliotibial band syndrome	2 (4.4%)
Bone overuse	1 (2.2%)

**Table 3:** Trunk accelerometry-based measures for participants that sustained an injury compared to the uninjured group at baseline i.e., during the 10–20% of the run. Values are means ± SD.

Parameters	Direction	Injured	Non-Injured	*p* Value	Partial Eta
*Spatio-temporal*					
Contact time (s)		0.22 ± 0.03	0.21 ± 0.02	0.38	0.006
Step frequency (steps/min)		170.8 ± 9.3	170.9 ± 7.9	0.82	0.00
*Dynamic stability*					
RMS ratio (unitless)	VT	0.83 ± 0.05	0.81 ± 0.04	**0.044**	**0.031**
	ML	0.43 ± 0.06	0.44 ± 0.06	0.33	0.007
	AP	0.34 ± 0.06	0.36 ± 0.06	**0.027**	**0.037**
Step regularity (unitless)	VT	0.93 ± 0.03	0.93 ± 0.02	0.27 #	0.02
	ML	0.69 ± 0.09	0.69 ± 0.10	0.97	0.00
	AP	0.72 ± 0.09	0.73 ± 0.09	0.62 #	0.00
Stride regularity (unitless)	VT	0.94 ± 0.02	0.95 ± 0.02	0.32 #	0.007
	ML	0.80 ± 0.06	0.79 ± 0.06	0.45	0.004
	AP	0.78 ± 0.07	0.77 ± 0.07	0.24	0.010
Sample entropy (unitless)	VT	0.08 ± 0.02	0.08 ± 0.02	0.82	0.00
	ML	0.15 ± 0.03	0.16 ± 0.03	0.097	0.021
	AP	0.16 ± 0.04	0.16 ± 0.03	0.69	0.001
*Dynamic loading*					
Impact acceleration (m/s^2^)	VT	3.61 ± 0.93	3.37 ± 0.96	0.15	0.016
	ML	2.32 ± 0.64	2.28 ± 0.62	0.67	0.001
	AP	2.20 ± 0.59	2.43 ± 0.57	**0.011**	**0.048**

# Based on the nonparametric Mann-Whitney U test. Significant differences between groups have been highlighted in bold. VT, vertical, ML, mediolateral, AP, anteroposterior.

## Data Availability

The data presented in this study are available on request from the corresponding author.
